# Nationally representative programmatic surveillance of mosquito and human behaviours that influence human exposure to malaria transmission and the impact of vector control across Tanzania

**DOI:** 10.1186/s12936-026-05893-1

**Published:** 2026-03-28

**Authors:** Maneno E. Baravuga, Praise Michael, Fadhila Kihwele, Brian Masanja, Selemani Mmbaga, Amos T. Mlalwe, Isaac Haggai Namango, Alanna-Jo McCallum, Nosrat Mirzai, Charles D. Mwalimu, Samwel Lazaro, Gerry F. Killeen, Samson Kiware, Heather M. Ferguson, Nicodem J. Govella

**Affiliations:** 1 Department of Environmental and Ecological Sciences, Ifakara Health Institute, Dar Es Salaam, Tanzania; 2 Wellcome Sanger Institute, Cambridge, UK; 3 Department of Epidemiology and Public Health, Swiss Tropical and Public Health Institute, Allschwil, Switzerland; 4 School of Biodiversity, One Health & Veterinary Medicine, College of Medical, Veterinary & Life Sciences, University of Glasgow, Glasgow, UK; 5 Bioelectronics Unit, University of Glasgow, Graham Kerr Building, Glasgow, UK; 6 National Malaria Control Programme, Dodoma, Tanzania; 7 School of Biological Earth and Environmental Sciences, University College Cork, Cork, Republic of Ireland; 8 Sustainability Institute, University College Cork, Cork, Republic of Ireland

**Keywords:** Vector surveillance, Human behaviours, Malaria vector behaviour, Vector control, Entomology, Outdoor exposure, Malaria transmission

## Abstract

**Background:**

Integrated entomological and anthropological surveillance of interacting mosquito and human behaviours is critical for understanding malaria transmission risks and for tailoring intervention packages, but it is yet to be implemented at scale due to a lack of practical, affordable procedures for nationally representative monitoring. This study in Tanzania introduces and field-tests the first such scalable framework design and reports summaries of the earliest data generated, thus demonstrating its feasibility and utility for generating programmatically informative indicators of both mosquito and human behaviours.

**Methods:**

A single village was randomly selected from each of twenty-five ecologically and epidemiologically diverse sentinel districts distributed across Tanzania for simultaneous surveys of malaria vector and human behaviours, using a rolling cross-sectional design. Entomological and anthropological surveys were repeated approximately annually over three years, with each village surveyed on three separate occasions across wet and dry seasons to account for temporal variations between sites due to seasons. Mosquito electrocuting traps (MET) were employed for sampling mosquitoes both indoors and outdoors with human behaviour surveyed by standard questionnaire.

**Results:**

Entomological and anthropological indicators were successfully and simultaneously measured in all 25 sentinel districts*. Anopheles arabiensis* was widespread across the country, whilst *Anopheles gambiae* s.s. and *Anopheles funestus* s.s. were more localized. Despite being collected in small numbers, blood-fed *An. funestus* s.s. and *An. gambiae* s.s. were predominantly human-fed, whereas *An. arabiensis*, *An. quadriannulatus* and *An. leesoni* fed predominantly on cattle. Higher proportions of host-seeking *An. arabiensis* were caught outdoors (64.9%) than *An. gambiae* s.s. (58.4%) or *An. funestus s.s.* (42.4%). Across all age groups, people were more likely to be outdoors during the early evening hours (18:00 to 21:00), with the highest proportions observed among adolescents (13–17 years) and adults (≥ 18 years) compared to under-fives (0–5 years) and children (6–12 years). Adolescent males were less likely to use bednet relative to other demographic groups. Higher temperatures were associated with reduced reported bednet use during the dry season but not during the wet season.

**Conclusion:**

This national surveillance framework proved highly effective for measuring relevant metrics of mosquito and human behaviours that can inform optimization of malaria control strategies.

## Background

Like many African countries, Tanzania has achieved significant reductions in malaria burden in recent decades. Since 2008, malaria prevalence has progressively reduced from a national average of 18% to 8.1% in 2022 [1]. Vector control tools, especially, the widespread distribution of insecticide-treated nets (ITNs), and to a lesser extent, indoor residual spraying (IRS), have accounted for most of this reduction, with enhanced access to effective antimalarial drugs and rapid diagnostic tests (mRDT), also contributing [2]. Despite these impressive gains and sustained community-wide use of ITNs, new malaria cases and deaths continue to be reported across Tanzania [3]. Malaria transmission is heterogeneously distributed across the country ranging from 0 to over 30% prevalence [1, 4–6]. Unfortunately, the underlying drivers of variation in transmission intensity and responsiveness to various control measures remain unclear. The lack of comprehensive understanding of the drivers and corresponding opportunities to address the problem may limit the ability of the National Malaria Control Program (NMCP) to effectively design and implement impactful, locally tailored interventions across diverse epidemiological and ecological settings [4]. Geographic variation in malaria risk can arise from differences in the human and mosquito vector behaviours that combine to determine exposure risk [7]. Thus, it is necessary to have effective surveillance systems that need to not only inform the malaria infection burden but also capture both types of exposure metrics (human and mosquito) simultaneously to identify transmission drivers and guide how vector control interventions could be tailored to local conditions to maximize impact.

Responding to these challenges, Tanzania, through the NMCP, adopted a subnational tailoring strategy [8]. This strategy aims to optimize intervention packages according to epidemiological burden (at district level) to maximize impact and ensure the rational use of limited resources [9–12]. For example, the current practice in areas with very low malaria prevalence ≤ 1%, mass distribution of insecticide treated nets (ITNs) is no longer a priority [13]. Instead, intensified case-based surveillance response systems are recommended in these settings, with larvae source management used as a supplementary control method [12] and/or reactive focal IRS combined with distribution of ITNs only to vulnerable groups (pregnant women and infants) [8]. While this major strategic re-orientation of malaria control in Tanzania from a “one-size-fits-all” approach to a stratified strategy has resulted in rapid reduction in high malaria burden regions, declining from seven in 2020 to only two in 2024, its full success will depend upon not only the surveillance systems that inform the malaria infection burden [4, 10] but should also integrate those that determine transmission drivers and how vector control interventions could be tailored to local conditions to maximize impact [7, 14–16]. This is particularly important because Tanzania is exceptionally diverse in terms of environment, ecology, livelihoods and human behaviours. It is therefore unrealistic to expect that a single optimal intervention will achieve the same impact across the country.

Currently, Tanzania relies on two malaria epidemiological surveillance systems: the Tanzania Demographic and Health Survey and Malaria Indicator Survey [1], an active cross-sectional country-wide survey which obtains human malaria infection data from under-five children, and is conducted every 4 years; and the School Malaria Parasitological Survey, which is also, an active survey but focusing on school age-going children. This survey is conducted every 2 years [10]. While these platforms are useful for informing malaria infection burden and distribution, they do not provide insights into the underlying causes (entomological and anthropological behaviours) of persisting transmission or how vector control should be optimized to maximize impact [15, 17–20].

In addition to these two epidemiological surveillance platforms [1, 10], the Tanzania NMCP implements a nationally representative longitudinal, community-based entomological surveillance system. This system involves monthly collections of data conducted over three consecutive nights in 32 sentinel districts across the country. It monitors vector densities, infection rates and species composition of malaria vectors [21]. Although this national platform provides useful entomological indicators, it is not sufficient by design to guide optimal selection of vector interventions. Specifically, such indicators cannot address the key questions such as what ecological and behavioural factors (mosquitoes and humans) limit the effectiveness of existing vector control interventions [22–24], or identify which combinations of existing or emerging vector control interventions could have the greatest impact within existing resource envelopes [15, 25].

While insecticide resistance monitoring is conducted annually and has confirmed widespread resistance across the country [26, 27], some of the most fundamental limitations of ITNs effectiveness could be behaviours of mosquitoes and humans that allow vector populations to survive by feeding at times when people are active outside of ITNs [23, 28–30], feeding outdoors [23, 31], and by feeding on animals [32]. However, these evasive behaviours also create opportunities to address them with complementary and potentially high impact measures, such as spatial repellent vapour emanators [33], which target mosquitoes when attacking human outdoors or at times when people are active outside of ITNs indoors, or endectocides for targeting mosquitoes when attacking livestock [34, 35]. It is, therefore, essential to incorporate measurements of behaviours of adult malaria-transmitting mosquitoes (such as biting time and location, and what mosquito feed on) and humans (such as time spent outdoors in the evening before retreating indoor to sleep and use of ITNs) into such programmatic surveillance platforms and assess how these behaviours are shaped across range of ecological settings. However, these types of data have never been previously surveyed at nationally representative level in Tanzania or anywhere else.

This study developed and implemented a first-of-its-kind, nationally representative survey that integrates intervention-targetable behaviours of malaria vectors and humans across ecologically distinct areas of mainland Tanzania. The data presented here are preliminary, mainly descriptive, based on pooled analysis rather than site-specific analysis. The aim is to provide an overview and demonstrate the feasibility and practicality of this framework for integrating programmatically useful behavioural indicators of both mosquitoes and humans within large-scale programmatic settings. Intensive spatial analysis accounting for interactions of species-specific malaria vectors and human behaviours will be presented in the further publications after completion of amplicon sequencing of mosquitoes which has not yet been completed.

## Methods

### Study area and sentinel districts

Mainland Tanzania is among the most environmentally and ecologically diverse countries in Africa. Located on the equator, it encompasses an Indian Ocean coastline and both branches of the Great Rift Valley. The country features diverse elevations ranging from sea level to over 2000 m, with marked variability in rainfall patterns. For example, the northern and north-eastern regions exhibit a short and long rainfall pattern, in contrast to the western and southern regions, which are characterized by only one long rainfall pattern in a year [36].

Malaria is endemic in Tanzania [37], with the malaria burden varying greatly across geographical areas [4, 6, 10]. *Anopheles arabiensis*, *Anopheles gambiae* s.s., and *Anopheles funestus* s.s. are the primary malaria vectors in Tanzania [21, 38–40]. *Anopheles arabiensis* is the most abundant and widely distributed across the country, while *An. gambiae* s.s. and *An. funestus* occur in relatively low numbers [21]. Despite their generally low abundance compared to *An. arabiensis*, *An. funestus* appears to exist in relatively high proportions in areas characterized by high malaria transmission [21].

**Fig. 1:**
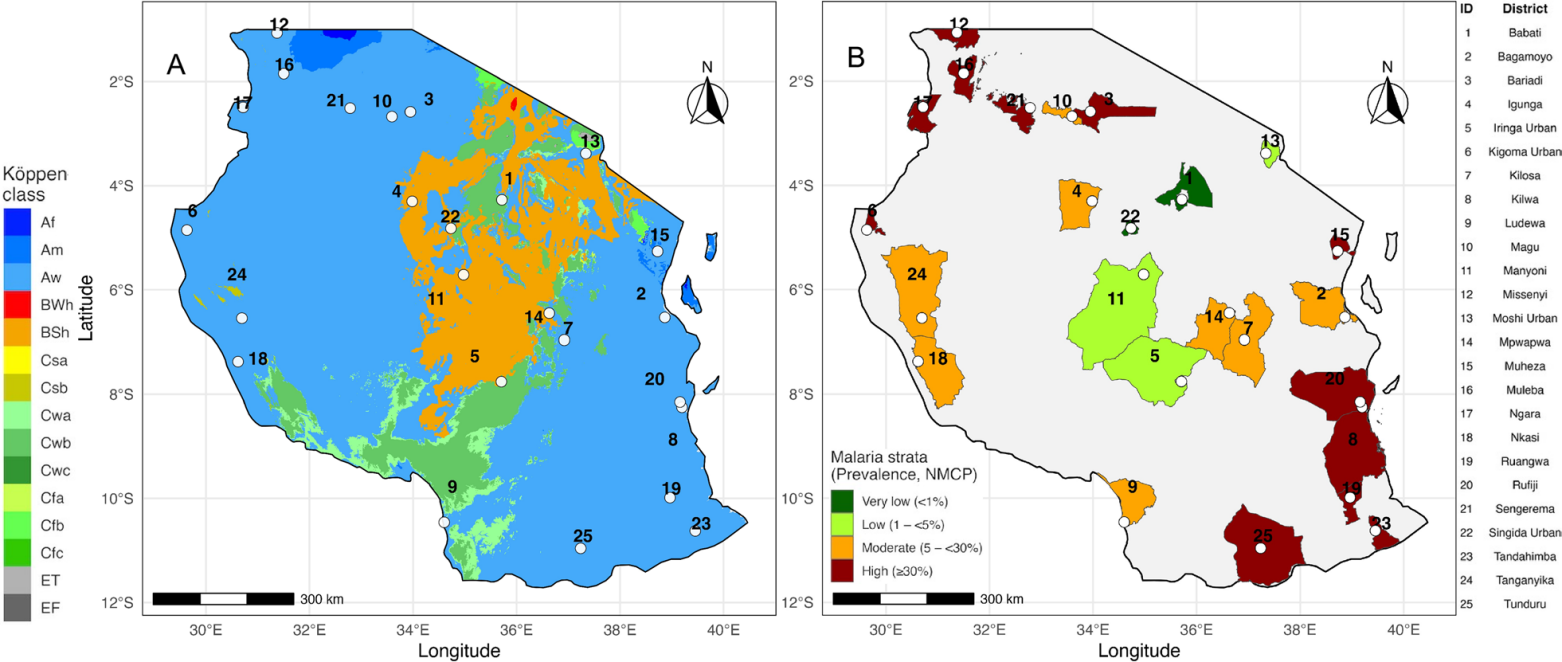
Climatic context of study sites (**A**) matched to distribution of sentinel districts by malaria risk strata (**B**). **A** Locations of mosquito sampling sites (white dots) overlaid on the Köppen–Geiger climate classification map of Tanzania. Tropical climates include Af (tropical rainforest), Am (tropical monsoon) and Aw (tropical savanna); arid and semi-arid zones include BWh (hot desert) and BSh (hot semi-arid steppe); temperate zones include Cfa (humid subtropical), Cwb (temperate oceanic with dry winters) and Cwc (subtropical highland with dry winters); polar climates are represented by ET (tundra, near the summit of Mount Kilimanjaro) and EF (ice cap conditions at the summit of Mount Kilimanjaro). **B** Distribution of sentinel districts according to national malaria transmission strata (Very low, Low, Moderate and High) overlaid by specific sampling sites (white dots). Sentinel districts are labelled with black numbers

As detailed above, in 2015, Tanzania NMCP established a network of 32 sentinel districts for routine monitoring of species composition of malaria vector species, mosquito densities and intensity of transmission [21]. From these, 25 sentinel districts, were selected for the survey on intervention-targetable behaviours of mosquitoes and humans (Fig. 1). The selection of sentinel districts was based on criteria that included: a diversity of malaria endemicity [4]; ecological diversity (e.g., highland vs. lowland, dry vs. wet areas, Great Lakes areas vs. coastal areas); operational diversity (e.g., irrigation vs. non-irrigation and urban vs. rural); intervention diversity (e.g. history of IRS vs. non-IRS), and geographical diversity (including areas bordering other countries) as described previously [21].

### Study design

A single village (out of the three villages under the NMCP entomological surveillance system) from each of the 25 sentinel districts was randomly selected for the survey on malaria mosquito and human behaviours. Within each village, three sub-village administrative units were selected, with four houses randomly selected in each using the local-government’s household listing. Accompanied by an appointed village representative, the research team visited the selected households and requested participation. Eligible houses included those with open eaves or broken window screens that allowed mosquito entry, so as to enable comparable estimation of mosquito distribution between indoor and outdoor spaces, and that had at least two bedrooms and a living room so that the research participant (mosquito collector) could carry out overnight indoor collections in a separate space from sleeping residents. Thus, mitigating intrusion of privacy to the household’s occupants due to the presence of a research participant. Additional criteria included obtaining informed consent from the head of household.

A rolling cross-sectional (mosquito and human behaviour) survey was implemented. Each selected single village was sampled on 3 separate occasions of 1 week, with sampling weeks distributed at different times of the year over the course of 3 years. This design allowed for site-specific estimates of mosquitoes and human behaviour while accounting for temporal variations due to seasons (wet vs dry) and weeks of sampling. Each of the 25 villages was sampled for 1 week per year, with three days dedicated for mosquito sampling conducted in parallel with human behaviour surveys in each village. With this design a full round of sampling at all villages was completed in 1 year, with time in between the annual 25 weeks of dedicated sampling used for travel, data entry and submission of mosquito samples to the central laboratory located at Ifakara Health Institute (IHI).

An additional round of sampling in years 2 and 3 provided repeated sampling at the same sites, but at different times of the year. As mosquito population dynamics are sensitive to seasonal variation, care was taken to ensure that all villages were sampled at least once during the rainy and in the dry season across the 3-year period. This was done by randomly allocating villages to sampling weeks during the first year of the study, with stratified randomization of sampling weeks implemented in year 2, such that villages sampled during a wet month in year 1 were assigned to dry month in year 2. The same procedure was repeated in year 3. In each of the 3 selected sub-villages, 4 houses were selected for mosquito sampling. In each of the 4 houses, mosquito sampling was conducted indoors and outdoors on each night. One night was spent sampling in each sub-village, meaning it took three sampling nights to complete mosquito sampling in a village during each round. This yielded a total of 24 trap nights per village per round, totalling 72 trap nights per village over the study period. In each survey round, a new set of four houses in each sub-village was selected to expand the spatial coverage of sampling within the village and also reduce potential pseudo-replications arising from repeatedly sampling the same place. As described in detail below, human behaviour surveys were conducted in the same houses and on the same days as mosquito sampling.

Travel between districts and within districts was facilitated using a single four-wheel drive Land Cruiser car, which accommodated a research team of up to five individuals. This group included a team lead, an entomology lead, a social science lead, and an entomology technician. Necessary field supplies, including tents, chairs, a table, a field microscope, mosquito traps, and consumables for storing mosquitoes were transported in the field vehicle.

### Survey of mosquito biting behaviours

Entomological surveillance was conducted at selected household with the specific goal of measuring mosquito biting behaviour, in terms of the timing (hour) and location (indoor or outdoor) of human-host seeking mosquitoes. Traditionally, these behavioural outcomes have been assessed using the human landing catch method [41, 42], in which mosquitoes are captured upon landing on exposed limbs of volunteers. Although this method provides a direct estimate of biting exposure, it raises ethical concerns due to the potential exposure of participants to infectious bites [42, 43]. Consequently, here, a novel exposure-free method the MET [41, 44–47], which has been field-validated and reproduced similar metrics of human exposure to mosquito bites both indoors and outdoors [41] and across the entire night [41, 46, 48] was used. The MET was selected because, unlike the human landing catch (HLC) which is the gold standard for characterizing distribution of mosquito bites between indoors and outdoors and at time of night, it captures mosquitoes without exposing volunteers to potential infectious bites [45, 47]. The MET is composed of four polyvinylchloride (PVC) panel frames aligned with electrocuting surfaces, arranged to form a square cavity into which human volunteer legs are placed [45]. The trap is powered by 24 V battery direct current (DC), which is regulated to produce current–voltage combinations which are just sufficient to kill mosquitoes trying to pass through the wires, but without destroying the specimens. The power is supplied at low output, which is sufficient to kill mosquitoes on contact, but poses negligible harm (or may cause mild shock) if accidentally contacted by volunteers [49]. The inner surface of MET is lined with insulators to prevent contact between volunteers and charged wires. As an auxiliary component of the MET, a retractable aluminium frame was constructed to enclose the remaining parts of the volunteer’s body with untreated, mosquito-proof netting. This configuration ensured complete protection of volunteers from mosquito bites during participation in trapping as described elsewhere [45].

During mosquito trapping, the participants sat on chairs under nets, with the lower sections of their legs inside the MET. To assess the distribution of mosquito biting over time and between indoor and outdoor location, mosquito collections by MET were conducted hourly by adult male volunteers between 1800 to 0700 h. Toward the end of each trapping hour (the last 15-min of each hour), mosquitoes were removed from the trapping surface and those that had fallen onto the floor, allowing for hourly measurement of biting activity. After trapped mosquitoes were removed and placed into pre-labelled paper cups within this same 15-min window period, participants were also given a break period for refreshment (drinks and snacks). Each night of trapping was divided into two shifts, with different teams of volunteers: the first team from 18:00 to 00:00, and the second team from 00:00 to 07:00.

Each morning after nightly collection, all mosquitoes were morphologically identified using appropriate taxonomic keys [50], counted, labelled and recorded electronically using tablets [51]. All female mosquitoes morphologically identified as *An. gambiae* s.l., *An. funestus* group, or other secondary malaria vectors (e.g. *An. coustani*, *An. ziemanni*, *An. pharoensis*) were stored individually in Eppendorf tubes filled with silica gel. Subsamples of the primary malaria vectors were initially subjected to molecular analysis using polymerase chain reaction (PCR) [52] at IHI to confirm species identity. All specimens that could not be amplified by PCR, along with the remaining specimens including secondary malaria vectors were shipped to the Wellcome Sanger Institute, in the United Kingdom, for further analysis using the ANOSPP amplicon sequencing panel [53]. For this proof-of concept paper, only PCR-based mosquito species identification results are presented because amplicon sequencing panel analysis is yet to be completed [53].

Complementing biting time and locations behavioural phenotype derived by MET measurement of host-blood meal source by malaria vector was also conducted. This requires collecting recently fed mosquitoes for molecular identification of their blood meal with the MET not being appropriate for this as it primarily target host-seeking mosquitoes. This was obtained by sampling dispersing blood-fed mosquitoes using barrier screen interception traps (BS) placed outdoors [47, 54] and/or prokopack aspirators [55] inside houses or cattle shed to sample blood engorged mosquitoes. In sub-villages with common livestock, of the four houses per sub-village, used for host-seeking sampling, at least one house with a cattle shelter was deliberately selected. This approach was taken to ensure a representative sample of mosquito feeding behaviour in the presence of humans and alternative hosts. At these households with livestock one BS was placed between the main house and cattle shed and potential resting sites. Another BS was placed at the outskirt of the sub-village to allow detecting what mosquito may feed on most in the absence of humans.

### Survey of human behaviours

Along with the entomological surveys, questionnaires were used to assess aspects of human behaviour that influence exposure to mosquitoes during nighttime hours (such as time spent outdoors in the evening during the mosquito biting period and reported use of ITNs for protection). Questionnaires were conducted at 12 households per village during each mosquito collection round (36 households over three rounds of surveys). These households were selected from the household listing of the village as detailed above for those participating in mosquito collections. A structured interview (lasting approximately 30–45 min) per individual was conducted. Questionnaires were first administered to the head or de facto head of household present at the time of the interview upon receiving an informed consent. These respondents were asked about how much time members of the household spend outdoors in the evening, where and when they usually eat dinner (in or outside), where they stay after dinner before going to bed, and what time doors and windows are shut. Subsequently, all other consenting household members were asked what time they typically go indoors, go to bed, get out of bed and leave the house in the morning. The present analysis focuses on questionnaire-based human behaviour data collected alongside the entomological surveys. Individuals from each age group: adult (18 and above), adolescents (13–17), school age going children (6–12), and under-five years were targeted. Assent was obtained from adolescents members of the households participating in the household questionnaire survey.

### Climatic condition measurement

Estimates of daily temperature, humidity and wind-speed were collected for each day of mosquito sampling using a portable Weather station (ClimeMET 2000 Professional Weather Station) which was installed between 5 to 10 m away from nearby houses used for mosquito collection. Temperature and humidity were recorded both indoors and outdoors, while rainfall and wind speed were recorded outdoors only. This was meant to assess how weather variability affects behavioural outcomes that are associated with exposure to malaria vectors such as use of ITNs and distribution of biting between indoors and outdoors.

## Data analysis

Data analysis was primarily descriptive except for testing the effects of age, gender, season and temperature on reported bednet use. In this analysis, a generalized linear mixed effects model fitted with a binomial distribution with a logit link function was applied, with reported bednet use (Yes/No) as the response variable. Age, gender, season, and mean-centred temperature were first fitted as fixed effects, followed by inclusion of interaction terms between age and gender, and between season and temperature. The day of survey, household, and sentinel districts were controlled for in the model as random effects. From this point the term “bednet use” is used instead of ITNs in order to be precise based on the questionnaire survey which used the question “Did you sleep under a bednet last night?”.

## Results

### General overview of mosquito collections

A total of 25,663 host-seeking mosquitoes were collected from 25 sentinel districts. These were morphologically identified as *Culex* spp. n = 21,067 (82.08%), *Coquilettidia* spp. n = 1384 (5.39%), *Anopheles gambiae* s.l. n = 1161 (4.52%), *Anopheles funestus* group, n = 1038 (4.04%), *Mansonia* spp., n = 661 (2.58%), *Anopheles pharoensis,* n = 121 (0.47%), *Anopheles coustani* group, n = 106 (0.41%), *Aedes* spp., n = 87 (0.34%), *Anopheles squamosus,* n = 18 (0.07%), *Anopheles ziemanni,* n = 15 (0.06%), *Anopheles maculipalpis,* n = 3 (0.01%), and *Anopheles rufipes,* n = 2 (0.01%). The number of each morphologically identified *Anopheles* species from each of the sentinel sites are represented in Table 1.

**Table 1:** The number of each morphologically identified *Anopheles* species from each of the sentinel sites

District	*An. gambiae s.l*		*An. funestus s.l*		*An. coustani*		*An. maculipalpis*		*An. pharoensis*		*An. rufipes*		*An. squamosus*		*An. ziemanni*		District Total
	Indoor	Outdoor	Indoor	Outdoor	Indoor	Outdoor	Indoor	Outdoor	Indoor	Outdoor	Indoor	Outdoor	Indoor	Outdoor	Indoor	Outdoor	
Babati	12	8	4	2	3	13	0	0	12	15	0	0	0	0	0	15	84
Bagamoyo	3	1	1	0	0	0	0	0	0	0	0	0	0	0	0	0	5
Bariadi	11	17	0	2	6	7	0	0	1	1	0	0	0	2	0	0	47
Igunga	38	131	1	0	4	1	0	0	11	50	0	0	2	2	0	0	240
Kigoma urban	10	27	1	10	0	0	0	0	0	0	0	0	0	0	0	0	48
Kilosa	18	48	158	190	0	0	0	0	4	1	0	0	0	1	0	0	420
Kilwa	24	16	51	26	0	0	0	0	0	0	0	0	0	0	0	0	117
Ludewa	8	3	79	3	10	1	1	0	3	3	0	0	2	0	0	0	113
Magu	7	6	29	23	0	12	0	0	0	3	0	0	1	0	0	0	81
Manyoni	10	72	0	1	0	1	0	0	2	7	0	0	1	1	0	0	95
Missenyi	39	32	216	63	8	8	0	0	0	0	0	0	0	0	0	0	366
Moshi urban	2	9	0	0	0	0	0	0	0	0	0	0	0	0	0	0	11
Mpwapwa	2	22	4	4	0	0	0	0	0	0	1	0	0	0	0	0	33
Muheza	6	3	2	1	0	0	0	0	0	0	0	0	0	0	0	0	12
Muleba	8	15	2	7	1	0	0	0	0	0	0	0	0	0	0	0	33
Ngara	17	19	13	19	3	0	0	0	0	0	0	0	0	0	0	0	71
Nkasi	10	9	1	2	1	6	0	2	1	1	0	0	0	0	0	0	33
Ruangwa	3	15	3	3	0	1	0	0	0	0	0	0	0	0	0	0	25
Rufiji	86	37	0	0	0	1	0	0	0	0	1	0	0	0	0	0	125
Sengerema	8	38	4	1	2	5	0	0	1	2	0	0	2	3	0	0	66
Singida urban	17	44	0	0	0	0	0	0	1	2	0	0	0	0	0	0	64
Tandahimba	6	21	0	2	1	1	0	0	0	0	0	0	0	1	0	0	32
Tanganyika	10	10	0	0	0	8	0	0	0	0	0	0	0	0	0	0	28
Tunduru	72	131	71	39	2	0	0	0	0	0	0	0	0	0	0	0	315
Grand Total	427	734	640	398	41	65	1	2	36	85	2	0	8	10	0	15	2464

## Species composition and distribution of the major malaria vectors

**Fig. 2:**
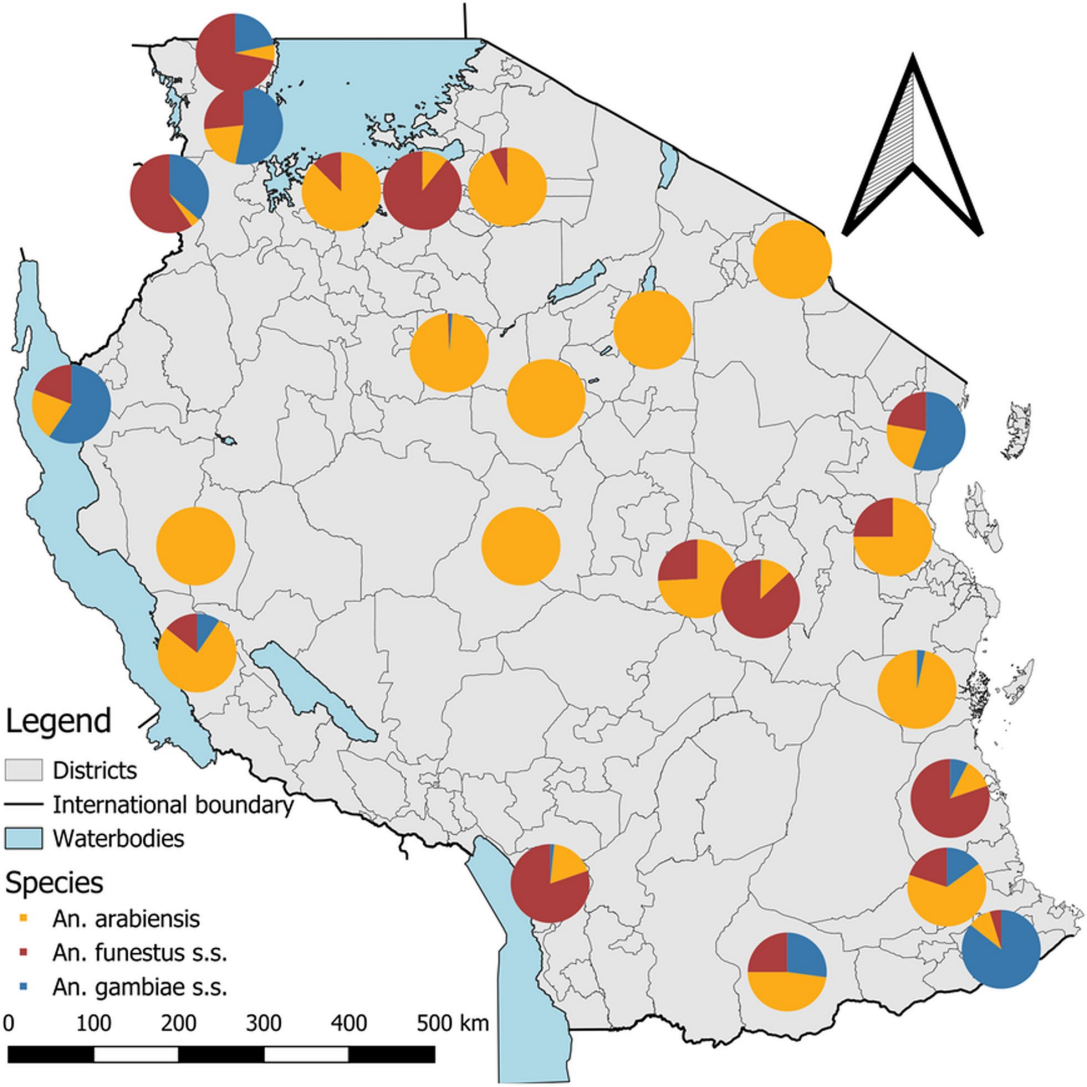
Species composition of the major malaria vectors and their distribution across sentinel districts

A total of 1749 out of 2199 mosquitoes from the *Anopheles gambiae* s.l. and *An. funestus* group were confirmed by PCR analysis representing 80% of the amplification rate. The amplified specimens consisted of *An. arabiensis* n = 762 (43.6%), *An. gambiae* s.s. n = 221 (12.6%), and *An. funestus* s.s. n = 766 (43.8%). *Anopheles arabiensis* was widespread across the country. In contrast, *An. gambiae* s.s. and *An. funestus* s.s. were more localized and were nearly undetectable in the relatively drier central corridor of the country (Fig. 2).

## Biting phenotypes of the major malaria vectors

**Fig. 3:**
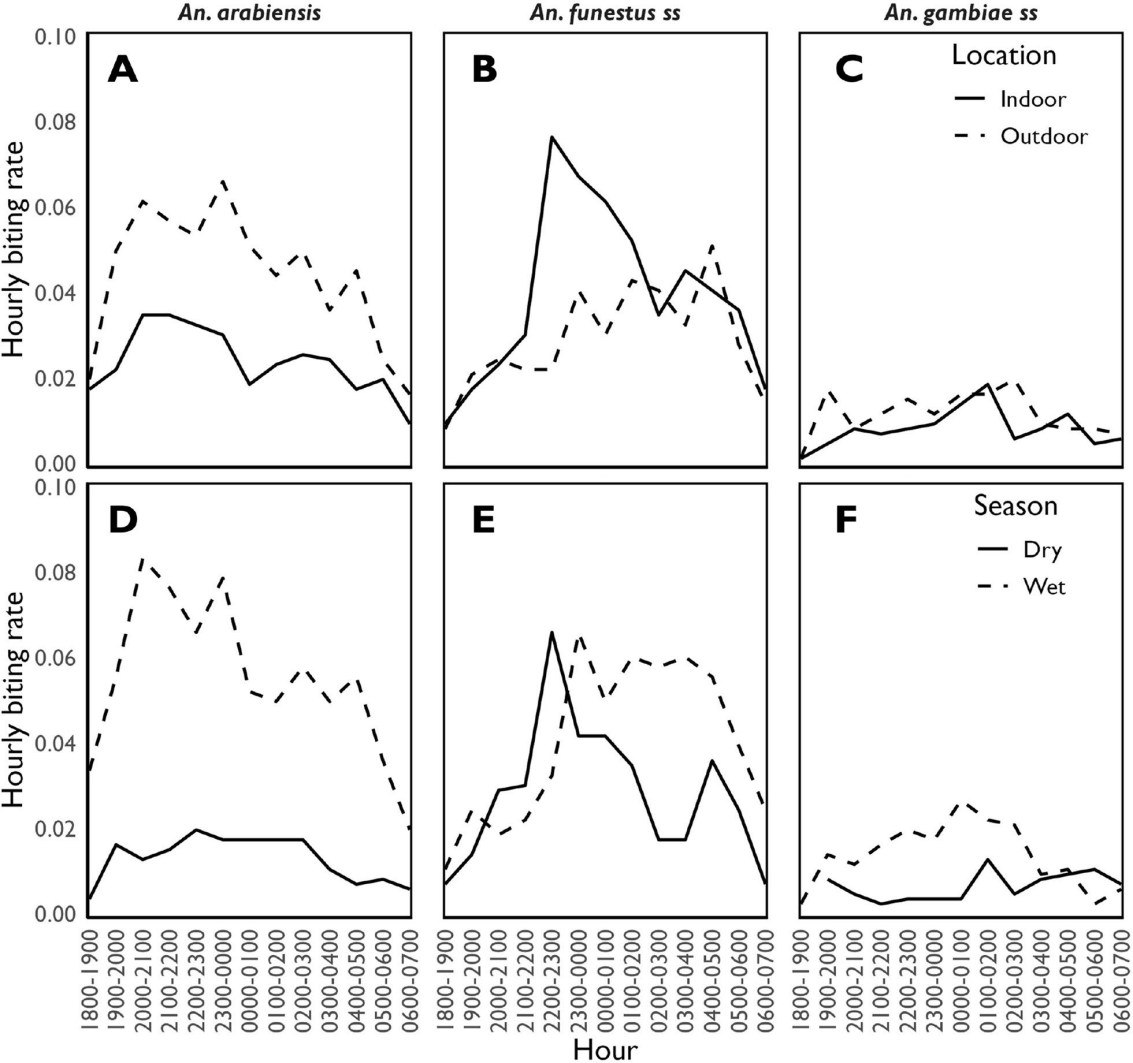
Biting location and time phenotypes of the major malaria vectors. Top panels (**A**, **B**, **C**) present a pooled analysis of biting location and time phenotypes regardless of the season, while bottom panels (**D**, **E**, **F**) show the analysis of biting time phenotypes stratified by season

A higher proportion of host-seeking *An. arabiensis*, were caught outdoors than indoors (outdoor = 64.9% versus indoor = 35.1%). In contrast, a higher proportion of *An. funestus* s.s., were caught indoors than outdoors (outdoor = 42.4% versus indoor = 57.6%). There was also slightly more *An. gambiae* s.s., caught outdoors than indoors (outdoor = 58.4% versus indoor = 41.6%), Fig. 3A–C. The biting activity for all the major malaria vector species started early in the evening between 18:00 to 19:00 h with *An. arabiensis*, reaching its first high peak between 20:00 to 21:00 h (for both indoors and outdoors), and second peak between 23:00 to 00:00, and biting persisted throughout the night with progressive decline between 04:00 to 07:00 h (Fig. 3A–C). In contrast, the first pronounced biting peak by *An. funestus* s.s. occurred between 22:00 to 23:00 h, with biting persisting throughout the night before progressively declining between 04:00 to 07:00 h (Fig. 3B). These observed biting time phenotypes were consistent between season (Fig. 3D–F). Both *An. arabiensis* (rainy season = 82.9% versus dry season = 17.1%) and *An. gambiae* s.s. (rainy season = 73.6% versus dry season = 26.4%) were substantially more abundant in the rainy season (Fig. 3D, F). However, seasonal variations were much less pronounced in *An. funestus* s.s., between season (rainy season = 58.7% versus dry season = 41.3%), (Fig. 3E).

## Measurement of host-blood meal sources in mosquitoes

The majority of blood-fed mosquitoes were collected using BS (n = 852, 93.2%), with smaller numbers obtained from the MET (n = 52, 5.7%), and the prokopack aspirators (n = 10, 1.1%). These specimens were analysed by ELISA to identify the vertebrate blood-meal sources present in the mosquitoes. Of the n = 914 female *Anopheles* specimens analysed, *An. arabiensis* comprised n = 834 (91.2%), *Anopheles quadriannulatus*, n = 39 (4.3%), *Anopheles leesoni*, n = 32 (3.5%), *An. funestus* s.s., n = 6 (0.7%), and *An. gambiae* s.s., n = 3 (0.3%). Blood-fed *An. arabiensis,* were collected from BS (n = 812), Prokopack aspirators (n = 6), and the MET (n = 16). Blood-fed *An. funestus* s.s. were collected from BS (n = 1), Prokopack aspirators (n = 4) and the MET (n = 1). All blood-fed *An. gambiae* s.s. (n = 3) and *An. leesoni* (n = 32) were collected using the MET, while all *An. quadriannulatus* (n = 39) were collected from the BS.

**Fig. 4:**
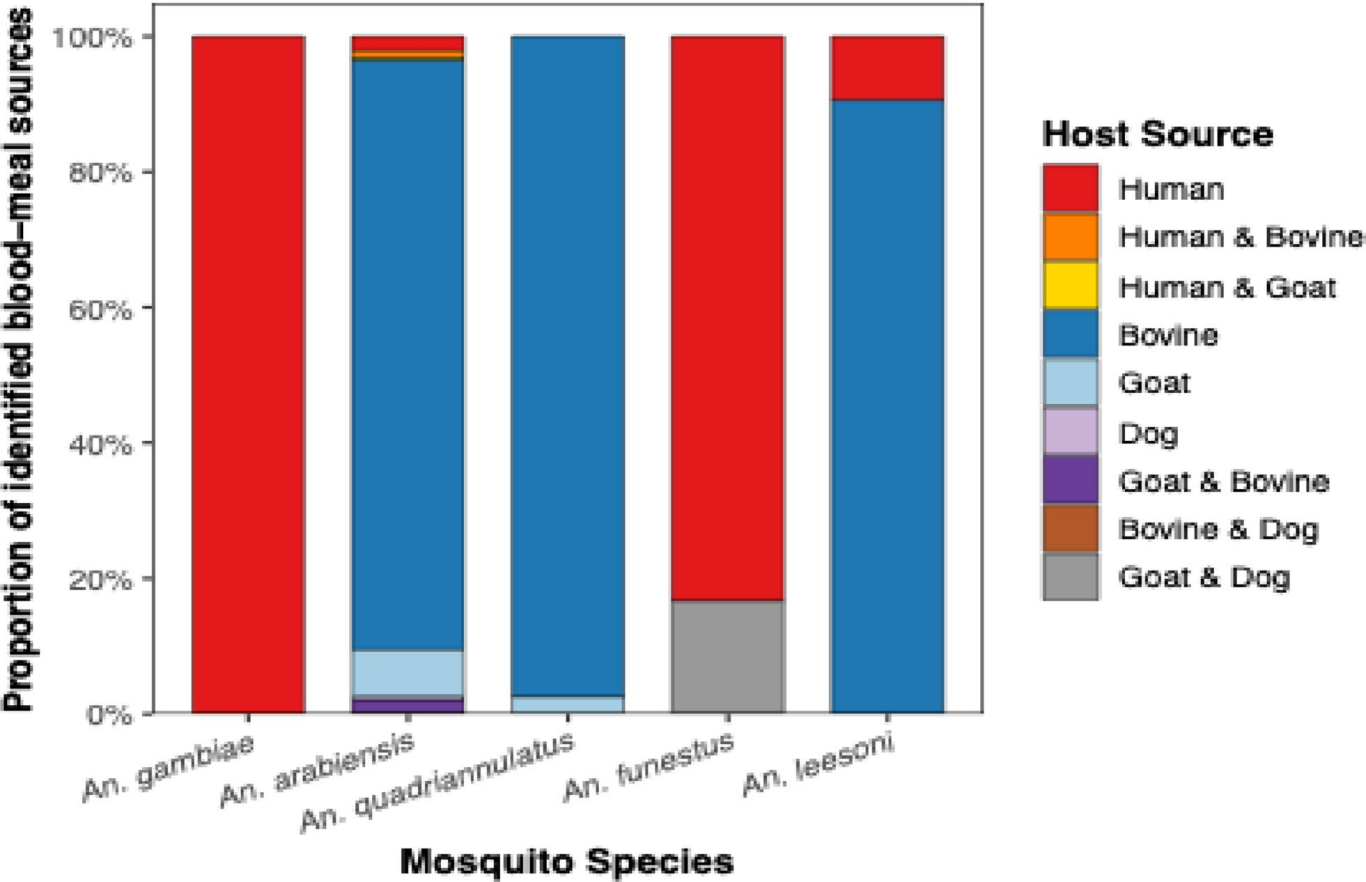
The distribution of host-blood meal sources across the *Anopheles* species

Figure 4 summarizes the distribution of host-blood meal sources across the *Anopheles* species. *An. arabiensis* fed on multiple host-blood meal sources, predominantly bovine n = 727 (87.2%), with smaller proportions feeding on goats, n = 58 (7.0%) and humans, n = 18 (2.2%). Mixed blood meal sources were detected in n = 33 (3.6%) specimens, consisting of combinations of human and bovine, human and goat or bovine and dog. Likewise, *An. quadriannulatus* fed predominantly on bovine, n = 38 (97.4%) with small fraction feeding on goats, n = 1 (2.6%). *An. leesoni*, also fed predominantly on bovine n = 29 (90.6%), with a minority feeding on humans, n = 3 (9.4%). Unlike these species, *An. gambiae* s.s., fed on humans, n = 3 (100%). *An. funestus* s.s., fed on humans, n = 5 (83.3%) and a mixed host-blood meal, n = 1 (16.7%) consisting of goat and dog.

## Human behavioural patterns across age groups

**Fig. 5:**
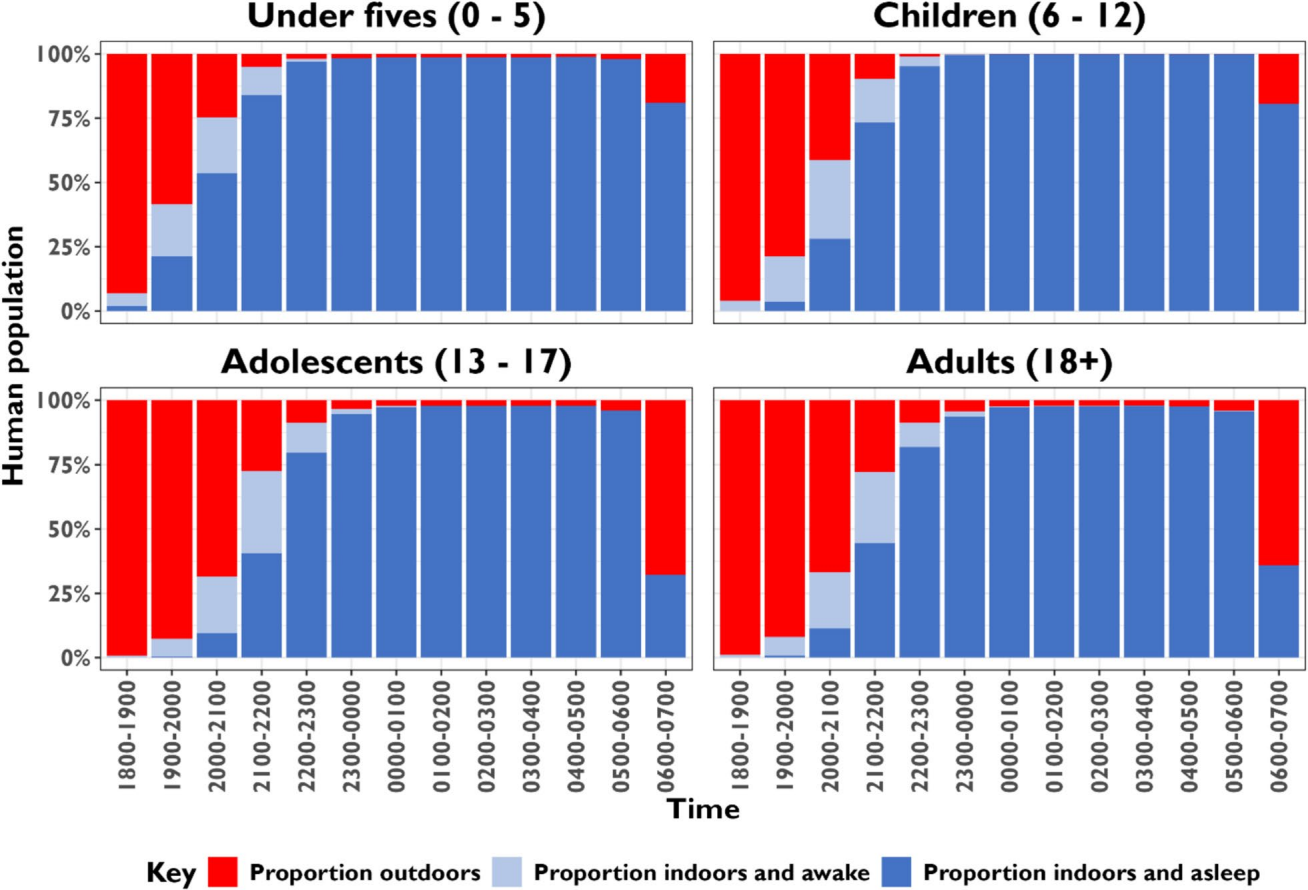
Hourly distribution of human behaviour between indoors and outdoors

Figure 5 illustrates the hourly distribution of individuals across three behavioural states associated with malaria vector exposure: outdoors, indoors and awake, indoor and asleep. Distinct age-specific patterns were evident. Time spent outdoor was concentrated during the early evening hours (18:00 to 21:00) across all age groups, with the highest proportions observed among adolescents (13–17 years) and adults (≥ 18 years) compared to under-fives (0–5 years) and children (6–12 years). Adolescents and adults remained outdoors later than younger age groups. Children aged 6–12 years and under-fives were predominantly indoors and transitioned to sleep earlier, generally between 20:00 and 22:00 h. More than 50% of the under-fives in a population were already indoors and asleep between 20:00 to 21:00 and proportion increased onward as opposed to only 6% of adults and adolescents during the same period. After midnight, nearly all individuals across all age groups were indoors and asleep until 06:00. Between 06:00 to 07:00 h, ≥ 65% of adults and adolescents were awake and outdoors. A small proportion of individuals (2–5%) after midnight remained outdoors throughout the night across all age groups.

## Determinants of the bednet use

**Fig. 6:**
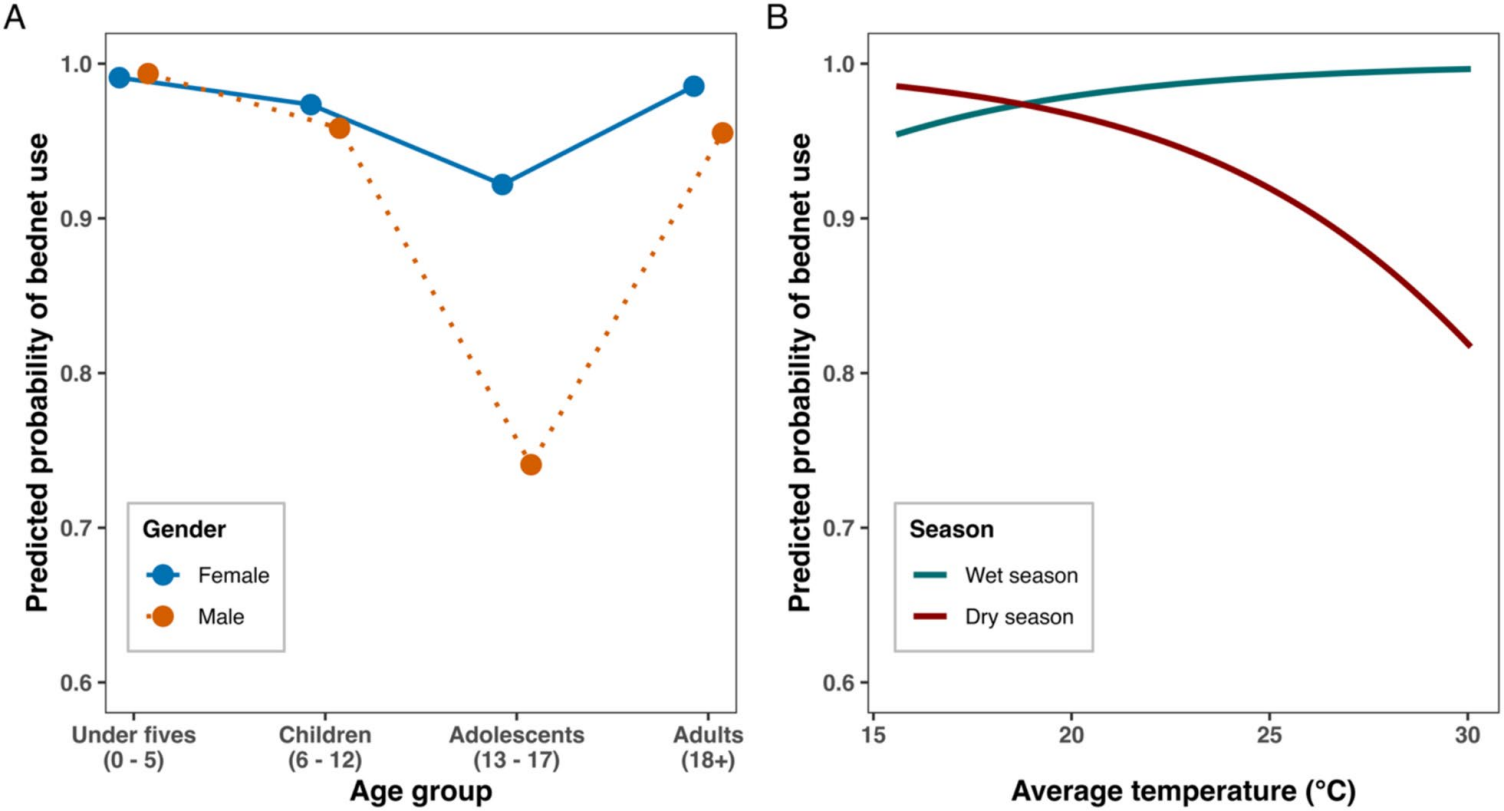
Predicted probability of bednet use from the binomial GLMM by **A** age group and sex, and **B** average temperature and season. **A** Predicted bednet use varied by age group and gender, with the largest difference between males and females observed among adolescents aged 13–17 years, consistent with a significant age-by-gender interaction. **B** Predicted bednet use remained high and relatively stable across temperatures during the wet season, but declined with increasing temperature during the dry season, consistent with a significant season-by-temperature interaction

Reported bednet use varied by the interaction between age and gender, and by the interaction between temperature and season (Table 2; Fig. 6). Compared with children under five years of age, children aged 6–12 years and male adolescents aged 13–17 years were less likely to report bednet use. Reported bednet use was also lower during the dry season at the mean observed temperature. Higher temperatures were associated with reduced reported bednet use during the dry season, whereas reported use remained high during the wet season regardless of temperature.

**Table 2:** Statistical summary of the generalized linear mixed model of reported bednet use, with age, gender, season, and temperature fitted as fixed effects, including interactions between age and gender and between temperature and season

Variables	Estimate	Standard error	Z-value	P-value
(Intercept)	5.3197	0.6104	8.716	< 0.001
Age Category: 13–17 years	− 2.2017	0.4755	− 4.630	< 0.001
Age Category: 6–12 years	− 1.0873	0.4447	− 2.445	0.014
Age Category: ≥ 18 years	− 0.4822	0.3614	− 1.334	0.182
Gender: Male	0.3485	0.5128	0.680	0.497
Season: Dry Season	− 1.5846	0.3913	− 4.050	< 0.001
Average Temperature	0.2228	0.1282	1.738	0.082
Interaction: 13–17 years x Male	− 1.7788	0.6796	− 2.617	0.009
Interaction: 6–12 years x Male	− 0.8332	0.6589	− 1.265	0.206
Interaction: > 18 × Male	1.5017	0.5768	− 2.604	0.009
Interaction: Dry Season x Average Temperature	− 0.4893	0.1573	− 3.110	0.002

The intercept represents females aged less than 5 years in the wet season at the mean observed temperature

## Discussion and conclusion

Based on these preliminary results, here, for the first time, we demonstrate the feasibility of simultaneously conducting entomological and anthropological surveillance, and the utility of this approach for nationwide surveys across Tanzania, which is critical for tailoring interventions. *Anopheles arabiensis* was widespread across mainland Tanzania, while the more efficient malaria transmitting primary vectors *Anopheles gambiae* s.s. and *Anopheles funestus* s.s., were observed to occur only in specific localized areas. *Anopheles arabiensis* exhibited a greater tendency for outdoor biting and with multiple blood-host choice compared to the other primary vectors. Consistent with the contribution of these primary malaria vector species to malaria transmission across Tanzania, a greater proportion of the blood-fed *An. funestus* s.s. and *An. gambiae* s.s. specimens contained human blood than non-human blood, despite the small numbers collected. In contrast, *An. arabiensis*, *An. quadriannulatus*, and *An. leesoni* fed predominantly on cattle. Barrier screen traps proved an efficient means to sample recently blood fed mosquitoes outdoors; a valuable sample for assessing human-biting tendency by different species which determine their relative transmission potential. Surveys of human behaviour conducted simultaneously with entomological data collection revealed that the majority of individuals including the under-fives, stay outdoors during early evening coinciding with time when substantial proportion of malaria vectors are also actively biting. Interestingly nearly all individuals reported being indoors and asleep after midnight, indicating that most malaria exposure during this time could be prevented by bednet use. However, a small proportion of the human population, including children under five years of age, were observed to remain outdoors throughout the night. Subsequent analysis of questionnaire data indicates substantial variation in reported bednet use being influenced by the interaction between age and gender and across temperature and season. This suggests bednet-based protection is far from uniform and quite variable between different populations. For example, male adolescents were less likely to sleep under bednet, compared to the children of under-five. Also, bednet usage decreased with increases in temperature during the dry season but remained relatively high during the wet season regardless.

The observed distribution patterns of the main malaria vectors align well with previous findings from the nationwide malaria vector surveillance system coordinated and managed by the NMCP in Tanzania [21]. The existing NMCP surveillance platform samples mosquitoes monthly using CDC light traps placed indoors in three villages per district, with a three-day sampling frequency. In the present study, comparable species composition and distributions metrics are reproduced using a more streamlined approach: sampling in only one village per district, with data collection stratified to include one occasion during the rainy season and one during the dry season. These results are not only encouraging but also suggest that the simplified design could be adapted to optimize the existing NMCP surveillance system, making it more cost-effective and relatively operationally feasible which is essential for providing a long term longitudinal entomological data collection platform.

While this design may not be necessarily perfect for distinguishing temporal from spatial variation in mosquito species composition and/ or behaviour, it reflects a balance between biological realism and programmatic feasibility, as demonstrated in this case. Importantly, the approach holds potential for application, addressing the growing concern that large-scale entomological surveillance is often prohibitively expensive. Adoption of this design could make nationally representative entomological surveillance both achievable and sustainable for malaria control programmes.

Despite the fact that majority of individuals reported to be indoors after midnight suggesting that most malaria exposure during this time could be prevented by bednet use, the persistent occurrence of humans outdoors at night particularly in early evening warrants considerable attention for sustaining malaria control gain. These quantitative estimates represent a fraction of the population at different time of the night at risk of malaria transmission outdoors, who cannot be practically targeted with conventional vector control interventions [2] due to their proximal circumstantial factors [16, 19, 24, 56–59]. Although this study was not accompanied by a qualitative survey to identify the specific circumstantial factors associated with nighttime outdoors presence, plausible explanations may include livelihood activities such as migratory farming [24, 60], security guards, cattle herders or other high-risk activities [24, 59, 61] and behavioural factors [22, 62]. While the proportion of people outdoors are generally relatively low from 22:00 h to 6:00 h compared to those indoors, these circumstances diminish the effective biological coverage of conventional malaria interventions [63] and may be sufficient to sustain residual transmission [7].

Sustained population wide use of ITNs remains critical [2], however, supplemental interventions targeting populations at risk in contexts where ITN use is not practical [23, 24, 59] should also be considered and promoted. While social and behavioural change communication strategies [64] may partly help to address this, practical experience indicates that it is often difficult to change people’s behaviours. Therefore, intervention tools specifically designed to accommodate or match the logistical challenges and/or behaviours posed by different circumstantial factors, a tailored delivery approach such as repellents [65, 66] may have appreciable impact.

Existing Tanzania NMCP malaria vector entomology surveillance system uses bucket traps [21] primarily to collect blood-fed mosquitoes for measuring mosquito host-choice, defined as the proportion of mosquito blood meals taken from humans relative to non-human hosts [32]. Host-choice is relevant in malaria programming because it determines the relative transmission intensity of different vector species and informs the selection of complementary or alternative control strategies [14, 67, 68]. It is widely established that *An. funestus* predominantly feed on humans, even in the presence of alternative hosts, whereas *An. arabiensis* exhibit flexible host feeding behaviours [46, 69]. These behavioural differences directly correspond to their relative contributions to malaria transmission [38], and their vulnerability to interventions [25, 70]. Accordingly, the occurrence of *An. funestus* s.s*.,* has been associated with high malaria burden strata compared with *An. arabiensis* [21]. Similarly, a previous entomological study in southern Tanzania showed that *An. funestus* s.s.*,* contributed approximately 80% of malaria transmission relative to *An. arabiensis* [38]. However, bucket traps have been shown to be ineffective at least at this scale of programmatic setting as evidenced in the previous NMCP report [21], where over five years of data collection (from 2017 to 2021), failed to generate reportable host-choice data. This study represents the first national-scale field evaluation of BS in Tanzania for measuring mosquito host-choice, and the results are consistent with previous reports showing that *An. funestus* s.s*.,* and *An. gambiae* s.s. are more likely to obtain blood meal from humans than *An. arabiensis* [46]. These results, however, should be interpreted with caution, particularly with respect to *An. funestus s.s.,* and *An. gambiae s.s.*, because fewer blood-fed individuals from these two species were collected and therefore may not be representative. However, the BS hold strong potential for large-scale characterization of host-blood meals by mosquitoes in outdoor settings. Additionally, BS are inexpensive, rely on locally available materials and are easy to use. These features are suited for programmatic use. Despite these advantages, the placement of BS in outdoor environments may bias collections toward mosquitoes that preferentially feed and rest outdoors, potentially leading to underestimation of the human blood index [32].

The notable variation in bednet use driven by overlap between age and gender and between temperature and season merit attention. Bednet-based protection is far from uniform and quite variable between different populations and environment. This suggests the need for behaviour change interventions tailored to specific demographic groups and times of the year to maximize the impact of bednet on malaria transmission. Growing evidence indicates that bednet use varies significantly by age and gender [71, 72]. This study suggests that the independent effects of age and gender may not be constant in determining use but variable. Here, interaction between age and gender appeared to be a key determinant of bednet use. Male adolescents were less likely to use bednet than their counterpart females and the rest of the demographic group. In particular, the behavioural risks associated with low utilization of bednet among male adolescents may partly explain the emerging epidemiological shift in malaria burden toward this demographic group [73–75].

While temperature appeared not to influence bednet use during the wet season [72], this may be because this period coincides with an increase in mosquito abundance, thereby motivating the population to use bednets consistently regardless of temperature [72]. A previous meta-analysis using data from 21 countries found that bednet use increased following peak rainfall periods which coincided with the increase in mosquito abundance [72]. The low utilization of bednets following increases in temperature during the dry season, is not surprising, but it highlights the concern, because this may contribute to sustaining perennial malaria transmission. For example, despite the overall reduction in biting densities of mosquitoes, this period coincides with the time where *An. funestus*, the widely known efficient malaria-transmitting mosquito species, exists in relatively high numbers as reflected in this study and other previous studies [38, 76, 77].

These earliest results are not without limitations. Although, the survey design allows for quantifying the overlap between human and mosquito behaviours across time and space to identify protection gaps in existing interventions and opportunities for complementary strategies [7], detailed mosquito species-specific analyses through amplicon sequencing [53] across sites remain incomplete. Species composition was inferred from the subset of samples that amplified successfully by PCR (80%). Although the species composition reported here aligns with previous studies, it may not represent the full diversity of species in Tanzania due to the existence of samples which could not be amplified by PCR. Additionally, the selection of houses with open eaves or broken window screens to allow mosquito entry in order to enable comparable estimation of mosquito distribution between indoors and outdoors, may not be ideal, particularly in urban settings where house architecture is changing rapidly. However, this could have been a fundamental limitation of this study if the design were intended primarily to assess transmission intensity. The survey design also did not incorporate formative (qualitative) questionnaires to explore underlying factors, such as motivations for spending time outdoors at night. The question about bednet use which was asked as “*Did you sleep under a bednet last night*” limited the ability to clearly understand the type of nets available in the population which are essential in assessing the effectiveness of ITNs. It remains unclear whether the reported behaviours reflect actual practices. Addressing this design gap is recommended to make the survey more responsive in guiding rational optimization of control strategies by national programs.

Despite these limitations, this first-of-its kind national surveillance framework proved feasible and holds potential for simultaneously measuring key metrics of mosquito and human behaviours, enabling identification of protection gaps and informing optimization of malaria control strategies at a programmatic scale.

## Data Availability

Access and use of data supporting this article may be made available upon receipt of official request through VBD360 Mosquito Database, (https://info.vbds360.io/projects) and it must ensure participants confidentiality and data privacy.
